# Complexities of Simultaneously Improving Quality and Lowering Costs in Hospitals: Comment on "Hospitals Bending the Cost Curve With Increased Quality: A Scoping Review Into Integrated Hospital Strategies"

**DOI:** 10.34172/ijhpm.2022.7442

**Published:** 2022-10-29

**Authors:** Dinesh R. Pai

**Affiliations:** School of Business Administration, Penn State Harrisburg, Middletown, PA, USA.

**Keywords:** Hospital, Cost Containmen, Quality Improvement, Value-Based Healthcare, Engagement, Health Information Technology

## Abstract

As health systems transition to value-based care delivery models, reducing costs and improving quality of care without sacrificing either remains a challenge for many healthcare organizations. There is extensive research on hospital costs, however, works addressing the complex relationship between hospital costs and the quality of care have been limited. In this commentary, I expound on the scoping review on integrated hospital strategies by Wackers et al that aim to improve quality while lowering costs. Specifically, I reiterate the complexity of the relationship between cost and quality and delve into major interdependent themes identified by the authors as relevant for the implementation of hospitals’ integrated strategy.

## Background

 Rising healthcare costs, variations in the quality of care, and access to healthcare have been of foremost concern to health policy-makers across the world. Health expenditure as a share of gross domestic product has increased from 8.8% in 2019 to 9.9% in 2020.^1^ The health expenditure also includes costly complications and unnecessary procedures due to poor quality of care mainly because of inefficient organization and management of medical care.^2^ Hospitals, which play a critical role in the delivery of healthcare services, are a significant component of the healthcare system across the world. For instance, the hospital component of health expenditure in the United States ($3.8 trillion) in 2019 was 31% or $1.2 trillion.^3^ Healthcare providers and policy-makers have focused on several initiatives to control cost and improve quality: Continuous Quality Improvement, a quality management strategy to improve processes and systems used to deliver care, and value-based healthcare, a strategy that focuses on quality of care which entails lowering healthcare costs or improving outcomes, or both. In a similar vein, several countries, such as Canada, Australia, and the United States (eg, Maryland and Pennsylvania), have implemented global budget programs to control hospitals’ spiraling costs and to improve the quality of care.^4^ The study by Wackers et al,^5^ which reveals key themes in implementing an integrated – high quality-low cost – hospital strategy, is timely and informs communities, payers, and hospital managers about the strategy, or the utility of other value-based payment models, in other settings.

 Wackers et al^5^ performed a scoping review to identify hospitals that have implemented high quality-low cost strategies and to determine factors influencing the adoption of these hospital-wide strategies. While many hospitals may target either of these goals – the high quality or low cost – separately and continuously, the authors argue addressing both goals within a single hospital-wide strategy may be more effective. The article adds to the scarce literature on hospital-wide improvement strategies by providing insights into the hospitals that have implemented an integrated strategy and on interdependent themes that were considered important during implementation. The authors extracted 265 relevant factors on integrated strategy implementation from the 19 cases that were reviewed. The factors were inductively categorized into 11 major themes: strategy, leadership, finances, engagement, projects, culture, support, reorganization, data collection, skill development, and communication. The lessons learned from the hospitals that employ a hospital-wide strategy of simultaneously increasing quality and reducing costs, and from the most addressed themes, could serve as a framework for the larger healthcare community. Given the increasing cost of healthcare and the expectations of higher quality of care, Wackers et al^5^ have addressed an important and a relevant topic in the article. The work contributes to the sparse literature on scoping reviews in general and to the ongoing debate on the cost-quality relationship in particular.

## Cost-Quality Relationship

 Wackers et al^5^ review cases on the cost-quality relationship in the context of the hospital-wide improvement strategy. Quality improvement in healthcare has been a growing concern ever since the Institute of Medicine’s landmark reports, “To Err Is Human” and “Crossing the Quality Chasm,” gained national attention and spurred extensive efforts devoted to measuring and improving quality.^6^ There is a broad consensus among policy-makers across the world on the need for improving quality and controlling costs. However, the relationship between quality improvement and cost reduction is complex.^7^ And, whether these two goals are complementary to or in competition with one another is yet unclear.^8^ Despite extensive research on hospital costs, there has been limited work examining the relationship between a hospital’s costs and the quality of care.^8^ Lack of reliable data on quality for most hospitals and consensus on appropriate measures of quality seems to have impeded the work.^8^ Healthcare quality is indeed difficult to define and quantify. And, characteristics such as the intangibility, simultaneity, and heterogeneity of hospital services make defining and measuring quality challenging.^9^ Wackers and colleagues’^5^ study adequately corroborates the heterogeneity of quality measures. In the 17 cases that report effects on one or more quality parameters, heterogenous quality measures such as length of stay, waiting times, avoidable readmissions, complications, scores on composite quality indices, patient satisfaction, and personnel outcomes were considered. Hussey et al,^10^ in their systematic review of US-based studies published between 1990-2012, found that the association between cost and quality is small to moderate, regardless of whether the direction is positive or negative. Furthermore, the relationship depends on clinical conditions and the specific resource utilization as well as on regulations for quality assurance in different countries and regions.^11^ For instance, Jha et al^8^ found low-cost hospitals in the United States had slightly worse performance on certain process-based quality indicators and comparable risk-adjusted mortality rates. Comparatively, empirical studies of the cost–quality relationship have received little interest among European hospitals.^7^ The complexity of the relationship and the plausible paucity of studies on the relationship partially explains the limitations such as selection, publication, and sample biases in Wackers and colleagues’^5^ study.

## Critical Factors Affecting Hospital-Wide Strategy

 Wackers et al^5^ identified 11 interdependent themes critical to implementing integrated strategies. These themes have the potential to affect successful implementation. For instance, successful implementation of electronic health records (EHRs), among other factors, depends on leadership, organizational culture, financial resources, and employee engagement. Wackers et al,^5^ therefore, urge hospital administrators to strike a balance across themes and detect potential conflicts. Among the hospitals that have implemented strategies to improve the quality of care, while containing or lowering costs, Wackers et al^5^ found data and information technology (IT), organizational strategy, and level of engagement as the most addressed key factors. We discuss below these key factors and their linkages to integrated strategies.

###  Data and Information Technology

 Wackers et al^5^ emphasize a data-driven approach to identify gaps in performance and potential cost savings. Furthermore, the authors emphasize investment in IT infrastructure to improve data collection efforts. The increasing focus on data-driven decision-making, spurred, at least partially, by legislative intervention and concomitant incentives, has led healthcare providers, including hospitals, in many countries to set up health information technology (HIT) infrastructure, which includes EHR. More recently, technologies such as telemedicine, mobile health apps, medical apps, and wearables have become increasingly popular among healthcare providers. The HIT infrastructure has led to a surge in health data generation, collection, storage, and analysis, creating opportunities to improve the quality of care, and patient experience and reduce healthcare costs. This data can be utilized to make data-driven decisions to provide better patient care and to improve hospital operations, essential ingredients for implementing integrated strategies. Predictive analytic models developed using artificial intelligence and machine learning help to predict patient prognosis and improve the health of individuals. For instance, demographic information and medical conditions of a patient presenting to an emergency department can help identify patients at risk for immediate undesirable outcomes after a fall by using machine learning.^12^ The increasing emphasis on value-based healthcare in recent years has led healthcare providers to focus on preventative and predictive measures concerning patient care, which is possible by leveraging analytics. Prior research suggests that HIT is positively associated with lower costs, and improved quality of care including lower readmission, reduced length of stay, and mortality rates,^13^ which is important for implementing integrated strategies. Despite the potential benefits, there are several barriers and challenges to the adoption and use of HIT: privacy and confidentiality concerns, lack of interoperability standards, insufficient data-sharing efforts, errors or delays in patient care due to mismatch of data transferred, lack of cooperation and consensus on data-sharing between competing providers, physician and organizational resistance, and information blocking, among others.^13^ There barriers, as noted by Wackers et al,^5^ impede data collection efforts or lead to incomplete data and reporting. As an illustrative case, the challenges to IT implementation and adoption bring to the fore the important role of engagement and organizational strategy can play in executing integrated hospital strategies, the other two key factors mentioned by Wackers et al.^5^

###  Role of Engagement

 Wackers et al^5^ have appropriately identified engagement as one of the key factors which is critical for implementing integrated strategies. The engagement of stakeholders, both within and outside a hospital, including patients, plays a critical role in executing an integrated strategy. A longitudinal study that explored the effects of employee (including clinicians, managers, and support staff) engagement in over 80 hospitals found greater engagement had a positive impact on outcomes such as hospital costs, treatment effectiveness (quality of care), and the level of hospital-acquired infections and conditions.^14^ Many hospitals have adopted employee (eg, physician and nurse) engagement – a critical factor for lowering costs while improving the overall quality of care – as a top strategic priority. For instance, discussions with IT practitioners have revealed that a bottom-up strategy that engages clinical staff (or end-user) is one of the key factors in successful EHR implementation. The practitioners informed EHR implementation has also enhanced communication with patients, provided access to patients’ medical records, and improved patient engagement and satisfaction. A recent scoping review list individual characteristics, work environment, and work outcomes as broad factors associated with physician engagement.^15^ Hospital leadership should develop strategies to improve work environment characteristics such as work-life balance, development opportunities, organizational support, autonomy, and social climate, among others. Perreira, Perrier, and Prokopy^15^ posit patient and staff safety, a culture of accountability among healthcare workers, and communicating evidence of the benefits and value of new practices as a few ways to improve worker engagement.

###  Organizational Strategy

 Hospitals are extremely complex and financially constrained systems, often exposed to political and regulatory vicissitudes. An organizational strategy that identifies clear priorities, engages all stakeholders, emphasizes teamwork, ensures effective communication, and has top leadership commitment is therefore important to improve the quality of care and lower costs. As hospitals wade through the challenges of cost-quality complexity, Wackers et al^5^ have identified several pertinent facilitators of, and barriers to, strategy themes. As stated by the authors, strategy is intrinsically linked to, and intertwined with, other themes. A bottom-up strategy, for instance, can help engage all stakeholders more effectively, get their support for important initiatives (eg, HIT implementation and concomitant data collection and dissemination efforts), and aid in skill development, among others. Some hospitals, however, disproportionately focus on cost reduction versus patient experience and outcomes, which can prove to be counterproductive, often leading to increased costs and at times lower quality of care. Indeed, as suggested by the authors, a focus on value creation by adopting value-based purchasing and developing an effective accountable care organization could help reduce costs and improve quality. As hospitals go about implementing simultaneous cost reduction and quality improvement initiatives, a simple strategy can help provide clarity in pursuing patient-centric, value-based approaches to patient care.

## Conclusion

 Using the scoping review method, a summative content analysis approach, Wackers et al^5^ address two important issues in healthcare: cost containment and quality improvement. Synthesizing available literature, the study identifies eleven interdependent themes influencing the simultaneous cost reduction – quality improvement strategies, or integrated strategies. The study has several limitations, such as selection, reporting, and publication biases, which may impede the generalizability of the results. Despite these limitations, however, the eleven themes could serve as a checklist for healthcare providers in general, and hospital managers in particular, to implement integrated strategies to obtain cost efficiencies and better patient outcomes.

## Ethical issues

 Not applicable.

## Competing interests

 Author declares that he has no competing interests.

## Author’s contribution

 DRP is the single author of the paper.
